# Hypoxia-Inducible Factor Signaling in Inflammatory Lung Injury and Repair

**DOI:** 10.3390/cells11020183

**Published:** 2022-01-06

**Authors:** Colin E. Evans

**Affiliations:** 1 Program for Lung and Vascular Biology, Section for Injury, Repair and Regeneration, Stanley Manne Children’s Research Institute, Ann & Robert H. Lurie Children’s Hospital of Chicago, Chicago, IL 60611, USA; colinevans@northwestern.edu; Tel.: +1-312-503-7466; 2 Department of Pediatrics, Division of Critical Care, Feinberg School of Medicine, Northwestern University, Chicago, IL 60611, USA

**Keywords:** injury, lung, pulmonary, regeneration, repair, vascular

## Abstract

Inflammatory lung injury is characterized by lung endothelial cell (LEC) death, alveolar epithelial cell (AEC) death, LEC–LEC junction weakening, and leukocyte infiltration, which together disrupt nutrient and oxygen transport. Subsequently, lung vascular repair is characterized by LEC and AEC regeneration and LEC–LEC junction re-annealing, which restores nutrient and oxygen delivery to the injured tissue. Pulmonary hypoxia is a characteristic feature of several inflammatory lung conditions, including acute lung injury (ALI), acute respiratory distress syndrome (ARDS), and severe coronavirus disease 2019 (COVID-19). The vascular response to hypoxia is controlled primarily by the hypoxia-inducible transcription factors (HIFs) 1 and 2. These transcription factors control the expression of a wide variety of target genes, which in turn mediate key pathophysiological processes including cell survival, differentiation, migration, and proliferation. HIF signaling in pulmonary cell types such as LECs and AECs, as well as infiltrating leukocytes, tightly regulates inflammatory lung injury and repair, in a manner that is dependent upon HIF isoform, cell type, and injury stimulus. The aim of this review is to describe the HIF-dependent regulation of inflammatory lung injury and vascular repair. The review will also discuss potential areas for future study and highlight putative targets for inflammatory lung conditions such as ALI/ARDS and severe COVID-19. In the development of HIF-targeted therapies to reduce inflammatory lung injury and/or enhance pulmonary vascular repair, it will be vital to consider HIF isoform- and cell-specificity, off-target side-effects, and the timing and delivery strategy of the therapeutic intervention.

## 1. Introduction

**Figure 1:**
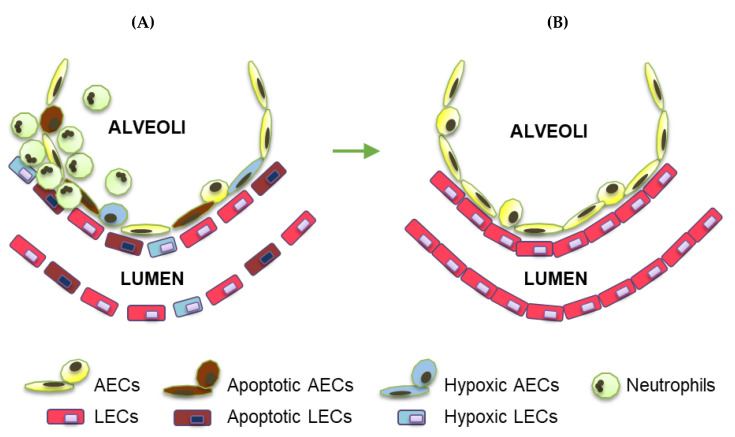
Characteristic features of inflammatory lung injury and repair. (**A**) Inflammatory lung injury is characterized by increases in LEC and AEC death, LEC–LEC junction weakening, and inflammatory cell infiltration. (**B**) Vascular repair following inflammatory lung injury is characterized by increases in LEC and AEC viability and proliferation, LEC–LEC junction re-annealing, and resolution of inflammatory cell infiltration.

Despite being generally well oxygenated, multiple pathological scenarios exist in which lung tissue becomes hypoxic [1]. Acute lung injury (ALI), acute respiratory distress syndrome (ARDS), and severe coronavirus disease 2019 (COVID-19) are examples of inflammatory lung conditions characterized by pulmonary hypoxia at the level of the tissue and cell. Triggers of lung injury that result in pulmonary hypoxia and alterations in lung function can be chemical (e.g., inhaled toxins) or mechanical (e.g., trauma). Common causes of ALI/ARDS include sepsis, pneumonia, smoke inhalation, and influenza or other viruses such as SARS-CoV-2. Other features of ALI/ARDS include apoptosis of alveolar epithelial cells (AECs) and lung endothelial cells (LECs) along with LEC–LEC junction weakening and leukocyte infiltration, which together result in vascular leakage and lung edema [2] (Figure 1A). In the chronic stages of lung injury and/or during situations of impaired vascular repair, reactive hyperplasia of AECs occurs and can lead to fibrosis [1]. In the reparative phase following pulmonary hypoxia and inflammatory lung injury, the lung repairs and regenerates via increases in AEC proliferation and LEC proliferation and junction re-annealing [3] (Figure 1B). Despite improvements in supportive care, there are currently no effective treatments for ALI/ARDS, and the mortality rate for ARDS is as high as 40% [2].

**Figure 2:**
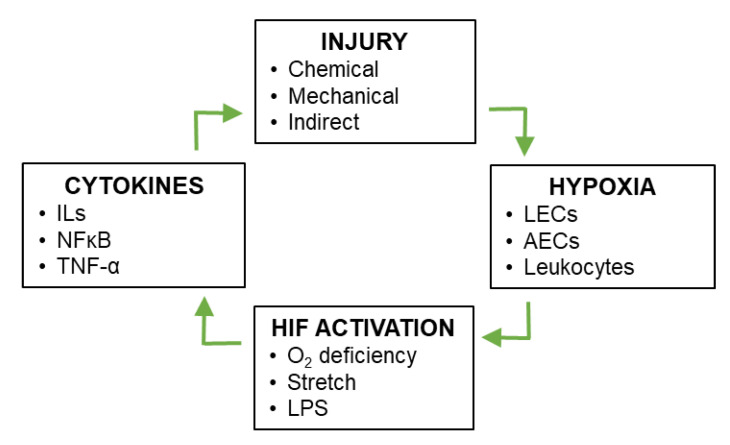
Positive feedback between vascular injury and HIF signaling during inflammatory lung injury. Pulmonary vascular injury leads to local conditions of hypoxia, which in turn leads to stabilization of HIF-1 and HIF-2 in multiple cell types of the lung. Downstream targets of HIF-1 and HIF-2 include anti-inflammatory and pro-inflammatory cytokines, the latter of which exacerbate inflammatory lung injury, creating a positive feedback loop.

During the pathogenesis of ALI/ARDS, damage to the lung vasculature causes hypoxia and defects in vascular permeability [4,5,6]. Levels of hypoxia are exacerbated in inflammatory lung by an imbalance between oxygen supply and demand; supply is disrupted due to alterations in vascular permeability and barrier function, while demand is increased due to increased metabolic requirements of the resident lung tissue and activated infiltrating inflammatory cells [7]. As explained in the following section, hypoxia leads to stabilization of the hypoxia-inducible transcription factors (HIFs). It has been suggested that various factors contribute to pulmonary HIF stabilization during ALI, including not only aberrant oxygen supply and demand, but also mechanical stretch and HIF stabilization via mediators such as inflammatory cytokines and lipopolysaccharide (LPS) [7]. HIF target genes include not only anti-inflammatory and reparative factors, but also pro-inflammatory factors such as including interleukin (IL)-1β, IL-6, tumor necrosis factor (TNF)-α, and nuclear factor (NF) κB [8]. These inflammatory factors further induce HIF activity [9,10,11] and exacerbate the severity of inflammatory lung injury, thereby creating a positive feedback loop (Figure 2). The aim of this review is to describe the regulation of inflammatory lung injury and repair by HIF signaling.

## 2. The HIF Signaling Cascade

The vascular adaptation to hypoxia requires a coordinated vascular response that is largely controlled by HIFs 1 and 2 [1]. HIF-1 is a transcription factor that was initially identified as a protein that binds to the hypoxia response element of the erythropoietin gene under conditions of hypoxia [12]. HIF-1 is highly conserved, widely expressed, directly regulated by oxygen availability, and regulates the expression of hundreds of target genes [13]. The HIF-1 heterodimer consists of two subunits, HIF-1α and HIF-1β. Under normoxic conditions, HIF-1β is constitutively expressed, but HIF-1α is ubiquitinated and targeted for proteasomal degradation by the von Hippel-Lindau (VHL) complex. However, conditions of hypoxia (e.g., 1% oxygen) enable the HIF-1α subunit to escape degradation and cause increases in HIF-1α protein levels and DNA-binding activity [14]. In other words, HIF-1α is the hypoxia-regulated subunit that controls the hypoxic induction of HIF-1-mediated gene transcription.

HIF-1α ubiquitination requires hydroxylation at two proline residues in human HIF-1α: Pro-402 and Pro-564 [15,16]. Under normoxia, HIF-1α hydroxylation is catalyzed by prolyl hydroxylase domain (PHD) enzymes 1–3, with molecular oxygen as a substrate [15,17,18]. PHD2 appears to be primarily responsible for HIF-1α hydroxylation in vivo [19,20]. Under hypoxia, PHD activity decreases, resulting in HIF-1α protein stabilization. HIF-1α then translocates to the nucleus, where it binds with HIF-1β to form the HIF-1 heterodimer. Coactivator proteins CBP and p300 are recruited to the HIF binding site within the hypoxia response element, which together activate the transcription of multiple HIF-1α target genes. A second HIF-α isoform, HIF-2α, is regulated by the same PHD-dependent degradation pathway and dimerizes with HIF-1β, but HIF-2α exhibits a more selective expression pattern that includes expression in LECs [21,22]. HIF-1 and HIF-2 target genes are distinct but overlapping and include factors that promote or inhibit vascular injury and repair. HIF target genes that directly regulate vessel barrier integrity include, but are not limited to, vascular endothelial growth factor (VEGF), VEGF receptor 1, and arginase [23,24]. The following section describes studies of the role of HIF signaling in inflammatory lung injury.

## 3. HIF-Dependent Regulation of Inflammatory Lung Injury

### 3.1. Systemic HIF Signaling

Experimental evidence from a mouse model of ALI induced by polymicrobial sepsis showed that hyperoxygenation increases mortality, while hypoxia reduces lung inflammation, suggesting that hypoxia-dependent signaling pathways are anti-inflammatory during ALI [25]. Studies of ventilation-induced lung injury in mice have implicated a role for HIF-1α in adenosine-induced lung protection during ALI [26]. Such studies support the idea of promoting HIF-1 signaling for the treatment of ALI/ARDS. However, circumstantial evidence from other studies suggests that HIF-1α can drive inflammation and ALI. For example, using a mouse model of allergic airway disease, Kim et al. found increases in the levels of HIF-1α that corresponded with increases in the production of inflammatory cytokines such as IL-4, IL-5, and IL-13, resulting in enhanced airway responsiveness and lung vascular permeability, which were reduced by systemic treatment with an agent that inhibits HIF-1 signaling [27]. In a study of LPS-treated rats, systemic treatment with emodin ameliorated ALI in parallel with reduced levels of inflammatory cytokines, and attenuated the expression of HIF-1α in whole lung tissues [28]. Emodin is a natural anthraquinone derivative that possesses anti-cancer, hepato-protective, anti-inflammatory, anti-oxidant, and anti-microbial properties [29,30]. In LPS-challenged mice, systemic treatment with tanshinone IIA reduced the production of inflammatory cytokines and decreased HIF-1α expression [31]. Similarly, propofol increased survival, reduced ALI, and decreased the expression of HIF-1α in whole lung samples from LPS-treated mice [32]. Propofol is a sedative-hypnotic agent that possesses anti-convulsant and broncho-dilatory properties [33]. Following treatment of rats with silymarin in a lung ischemia/reperfusion injury model, lung inflammation was suppressed, together with inhibited activation of caspase-3 and -9, and reduced HIF-1α protein expression [34]. Silymarin is a derivative of *Silybum marianum*, which possesses anti-oxidant, immune-modulatory, anti-fibrotic, anti-proliferative, and anti-viral properties [35,36]. Furthermore, there are positive feedback loops between HIF-1α and the expression of several inflammatory cytokines [9,10,11], and hypoxia worsens inflammatory ALI via toll-like receptor 4 signaling [37]. In mouse models of gut ischemia/reperfusion or trauma-hemorrhagic shock, partial HIF-1α deficiency attenuates resulting lung injury [38,39]. In a rat model of trauma-hemorrhagic shock, the HIF inhibitor YC-1 reduces resulting lung injury [40]. In a hypoxic ischemia/reperfusion model, increased levels of pulmonary HIF-1α protein are associated with increased VEGF levels and greater vascular disruption [41]. However, studies of systemic hypoxia or pharmacological therapies are limited in their ability to decipher the roles of cell-specific HIFs in inflammatory lung injury. As described below, cell-specific HIF signaling can regulate inflammatory lung injury and repair in a manner that is dependent upon the HIF isoform, cell type, and pulmonary insult in question. In other words, it seems that HIF signaling in different cell types can function differently under different pathologic conditions.

### 3.2. HIF Signaling in LECs

In a study of endotoxemia sepsis, endothelial cell (EC)-specific PHD2 depletion reduced lung vascular permeability, edema, and inflammatory cell infiltration [42]. EC-specific PHD2 knockout mice also exhibit enhanced adherens junction integrity and endothelial barrier function [42]. In isolated mouse LECs, PHD2 knockdown induced VE-cadherin expression [42]. In human LECs, hypoxia-induced increases in the expression of the inflammatory cytokine IL-8 were found to be dependent upon HIF-1α [43]. Although these studies indirectly suggest that LEC-specific HIF-1 could play both barrier-protective and barrier-disruptive roles in ALI, none of them directly assessed whether loss or gain of HIF-1α in LECs alters lung injury.

In human pulmonary microvascular ECs, exposure to septic lymph decreases EC viability and increases HIF-1α expression [44]. HIF-1α gene silencing in human pulmonary microvascular ECs increases cell viability and reduces inflammatory cytokine expression after incubation with septic lymph [44]. These results indicate that HIF-1α is induced during inflammatory EC injury and that inflammatory LEC injury occurs via HIF-1α [44]. In a rat model of burn injury, serum levels of HIF-1α increase to a peak at 12 h post-burn and are associated with increases in lung vascular permeability [45]. In isolated rat aortic LECs, increased HIF-1α expression leads to reductions in the expression of VE-cadherin [45], a key adhesion molecule located at the EC–EC junctions. Conversely, decreased HIF-1α expression results in increased VE-cadherin expression [45]. In another study of rat LECs, HIF-1α siRNA treatment reduced LEC permeability following hypertonicity and hypoxia [46]. In human pulmonary artery LECs, hypoxia-induced increases in LEC permeability are reduced by treatment with HIF-1α siRNA or an antagonist of the HIF-1 target, vascular endothelial growth factor (VEGF) [47]. These studies together suggest that LEC-specific HIF-1α increases EC permeability via reductions in VE-cadherin expression and increases in VEGF expression.

Regarding EC-specific HIF-2α signaling in mouse lungs, the EC-specific deletion of HIF-2α results in LEC loss and emphysema [48]. After exposure to Sugen SU5416, EC-specific HIF-2α knockout mice develop more severe emphysema, while mice with overexpression of EC HIF-2α are protected [48]. EC-specific HIF-2α knockout mice exhibit reduced levels of hepatocyte growth factor (HGF) [48], while human emphysema lungs also exhibit reduced expression of LEC-specific HIF-2α [48]. In a mouse study of lung ischemia/reperfusion injury [49], HIF-2α and β-catenin were downregulated in lung tissue and LECs following ischemia/reperfusion injury compared with uninjured lung tissue and LECs [49]. Further, miR-223 negatively regulated the expression of HIF-2α and β-catenin in lungs and LECs, while autophagy and apoptosis were increased by HIF-2α inhibition in LECs treated with a miR-223 antagonist [49]. HIF-2α inhibition also increased ischemia/reperfusion injury in mice treated with a miR-223 antagonist [49]. In another study of ischemia/reperfusion injury, knockdown of aquaporin-1 increased cell death (apoptosis and necrosis) and enhanced the permeability of pulmonary microvascular ECs—effects that could be partly rescued by HIF-2α overexpression [50]. These findings indicate that EC-specific HIF-2α prevents emphysematous and ischemia/reperfusion lung injury.

In a study of LEC junction integrity in mice following endotoxemia sepsis, vascular endothelial protein tyrosine phosphatase (VE-PTP) was found to be an HIF-2α target [51]. HIF-2α-dependent VE-PTP expression enhanced VE-cadherin dephosphorylation, which in turn reduced VE-cadherin endocytosis and augmented LEC barrier function [51]. In mice with EC-specific deletion of HIF-2α, VE-PTP expression was decreased, and VE-cadherin phosphorylation increased, resulting in defective LEC junctions [51]. Mice lacking EC HIF-2α exhibited increases in lung vascular permeability at baseline and following endotoxin-mediated lung injury [51]. Treatment of EC-specific HIF-2α knockout mice with a PHD2 inhibitor, FG-4497, increased VE-PTP expression, decreased VE-cadherin phosphorylation, and promoted LEC junction integrity [51]. In a study of murine airway microvasculature, EC-specific knockout of HIF-2α but not HIF-1α caused tracheal EC apoptosis, and reduced vascular perfusion, defective barrier function, and subepithelial fibrosis [52]. HIF-2α enhanced EC integrity in airways via EC angiopoietin-1/TIE2 signaling and Notch activity [52]. In tracheal transplants, EC-specific HIF-2α deficiency caused micro-vessel loss, whereas HIF-2α or angiopoietin-1 overexpression improved microvascular perfusion and integrity [52]. These findings together show that EC HIF-2α is required for airway microvascular health and that HIF-2α in LECs promotes LEC barrier integrity. Activation of HIF-2α signaling could therefore reduce ALI in lung inflammatory diseases such as ARDS and severe COVID-19. Experimental studies of endothelial dysfunction in other disease models (e.g., pulmonary hypertension models) have also demonstrated how EC-specific HIF-2α can regulate endothelial dysfunction and pathogenic remodeling [53,54,55,56].

### 3.3. HIF Signaling in AECs

In ventilator-induced lung injury, HIF-1α signaling in AECs has been shown to play a protective role [57]. In a study by Eckle et al., AECs were exposed to cyclic mechanical stretch, which stabilized HIF-1α expression, as did ventilator-induced ALI, even under normoxia [57]. Subsequent pharmacological studies using HIF activators or inhibitors revealed that HIF-1α stabilization attenuated pulmonary edema and lung inflammation during ALI, while treatment to inhibit HIF transcriptional activity resulted in increased susceptibility to lung injury [57]. The authors then carried out specific genetic deletions of HIF-1α in ECs, AECs, or leukocytes, and showed that the protection by HIF-1α against ALI was dependent upon AEC-specific HIF-1α [57]. A study by Zhao et al. also found that AEC-specific HIF-1α knockout mice developed greater lung viral replication, more severe lung inflammation, and increased mortality following influenza A infection [58]. In a separate study, Rosenberger et al. showed that HIF-1α-dependent induction of netrin-1 in AECs reduced hypoxia-induced inflammation [59]. These investigations reveal a role for AEC-specific HIF-1α in lung protection during ALI. In cultured AECs, however, Krick et al. demonstrated that hypoxia-induced increases in cell apoptosis are dependent upon HIF-1α [60], and He et al. showed that pharmacological down-regulation of HIF-1α leads to reductions in hypoxia-induced apoptosis [61]. These studies suggest that HIF-1α signaling in AECs drives AEC death.

Regarding HIF-2α signaling in AECs, Proper et al. hypothesized that AEC-specific HIF-2α activation contributes to the pathogenesis of ALI [62]. These authors assessed whether loss of HIF-2α in lung AECs protects against cobalt-induced inflammation; in their study, mice with HIF-2α deletion in club cells and type II AECs were compared with wild-type controls [62]. Results showed that conditional loss of HIF-2α enhanced inflammation and increased metaplasia, suggesting that AEC-specific HIF-2α plays an important role in ALI [62].

### 3.4. HIF Signaling in Leukocytes

In response to endotoxemia, myeloid HIF-1α appears to play a detrimental role, given that myeloid deletion of HIF-1α is protective against LPS-induced mortality and septic shock [63]. Peyssonnaux et al. showed that LPS increases the level of HIF-1α in macrophages, and decreases PHD production in a TLR4-dependent manner [63]. Using myeloid-specific HIF-1α knockout mice, these authors demonstrated that HIF-1α is a critical protagonist of the sepsis phenotype; HIF-1α promoted the expression of inflammatory cytokines including TNF-α, IL-1, IL-4, IL-6, and IL-12 [63]. HIF-1α deletion in macrophages protected against LPS-induced mortality and prevented the onset of hypotension and hypothermia [63]. Cramer et al. also found that HIF-1α deletion in macrophages reduced the production of the inflammatory cytokine TNF-α in response to LPS exposure with or without hypoxia [64]. In a separate study, Li et al. found that ventilation-induced AEC apoptosis and lung injury in mice is reduced by HIF-1α deletion in CD4^+^ leukocytes [65]. These studies suggest that the inhibition of HIF-1α in myeloid cells represents a potential therapeutic strategy for treating sepsis-induced ALI, but the role of leukocyte-specific HIF-2α in inflammatory lung injury is yet to be fully elucidated. In the following section, studies of the role of cell-specific HIF signaling in vascular repair following inflammatory lung injury are described.

## 4. HIF-Dependent Regulation of Lung Repair Following Inflammatory Lung Injury

### 4.1. HIF Signaling in LECs

HIF-1α is rapidly increased in murine LECs after endotoxemia sepsis challenge [66]. Mice with EC-specific knockout of HIF-1α exhibit impaired LEC regeneration and vascular repair in contrast to wild-type mice, despite similar levels of peak injury [66]. Overexpression of FoxM1 in the LECs of these EC-specific HIF-1α knockout mice normalized LEC proliferation and vascular repair, showing that LEC HIF-1α is required for LEC regeneration and vascular repair via FoxM1-mediated EC proliferation after sepsis-induced lung injury [66]. In another study of HIF-1α signaling during post-sepsis vascular repair in mouse lungs, promoter analysis identified Sox17 as a transcriptional target of HIF-1α [67]. Sox17 is a member of the family of SoxF transcription factors and a key regulator of endothelial and hematopoietic cell lineages [68]. Sox17 was induced in LECs 6 h post-sepsis in mice [67]. EC-specific knockout of Sox17 resulted in reduced LEC proliferation and persistently increased levels of pulmonary vascular permeability following endotoxemia challenge [67]. Conversely, Sox17 overexpression in LECs enhanced LEC proliferation and regeneration and promoted survival [67]. A recent study in our laboratory used high-throughput screening of a library of 1200 FDA-approved drugs to identify candidate therapies that enhance HIF signaling (Evans et al., submitted). In this study, rabeprazole (also known as Aciphex) resulted in dose-dependent increases in HRE luciferase activity. By treating mice with oral rabeprazole on two consecutive days after sepsis challenge, we next identified a dose of that was well tolerated and enhanced vascular repair and resolution of inflammatory lung injury. Rabeprazole gave rise to reductions in lung vascular leakage, edema, and inflammatory cytokine expression during the repair phrase post-sepsis. We also used conditional knockout mice to show that rabeprazole promotes vascular repair through HIF-1α and FoxM1. Thus, rabeprazole is one example of a clinically approved drug with potential for repositioning to treat ALI/ARDS. These studies together demonstrate the crucial role of LEC-specific HIF-1 signaling in LEC repair and regeneration following sepsis through increased FoxM1 and Sox17 signaling.

Apelin is another factor that has been identified as a reparative HIF-1 target, although the role of this proliferative factor in models of lung injury and repair are lacking [69]. In a mouse orthotopic tracheal transplant model, topical application of deferoxamine, which promotes HIF-1 signaling, resulted in increased LEC proliferation and decreased apoptosis [70]. In another study of airway microvascular regeneration, VHL-haplodeficient airway ECs exhibited enhanced microvascular repair, as shown by increased EC survival and migration [71]. Conversely, microvascular repair was impaired in transplant-recipient mice with HIF-1α deletion in Tie2-positive cells [71]. These studies together show the importance of EC-specific HIF-1α in airway vascular repair following lung transplant.

In terms of LEC-specific HIF-2α signaling, one study assessed aged mouse lungs following pneumonectomy, in which fibrosis was increased and alveolar regeneration was impaired compared with young mice [72]. These differences were associated with increases in the expression of neuropilin-1 and HIF-2α in the LECs of aged versus young mice after pneumonectomy [72]. In aged lung, induction of neuropilin-1/HIF-2α signaling in ECs inhibited the anti-thrombotic and anti-inflammatory endothelial protein C receptor (EPCR) pathway, which increased fibrosis [72]. Conversely, the EC-specific deletion of HIF-2α increased EPCR expression in aged LECs after pneumonectomy, while the genetic inhibition of EC-specific neuropilin-1/HIF-2α reduced fibrosis and restored regeneration in the lungs of aged mice treated with bleomycin [72]. In a mouse model of immunotherapy-induced pneumonitis, the EC-specific deletion of HIF-2α reduced mortality and lung fibrosis, which was reversed by treatment with EPCR antibody [72]. These studies together suggest that HIF-2α in LECs suppresses EPCR expression, which results in enhanced fibrosis and impaired lung regeneration. LEC-specific overexpression of HIF-2α increased proliferation under normoxia and hypoxia, while HIF-2α deletion in isolated LECs reduced normoxic and hypoxic proliferation [73]. Further studies are required to fully define the role of EC-specific HIF-2α in vascular repair following inflammatory lung injury.

### 4.2. HIF Signaling in AECs

In an AEC injury model induced by tracheal instillation of LPS or hydrochloric acid, epithelial HIF-1α has been shown to promote type II AEC proliferation and spreading for epithelial repair [74]. McClendon et al. hypothesized that HIF-1α promotes the proliferation and spreading of type II AECs during repair following lung injury. In their study, mice were treated with LPS or hydrochloric acid [74]. Post-injury HIF activation was demonstrated in type II AECs by increased luciferase activity in HIF reporter mice and increased expression of the HIF-1α target, GLUT1 [74]. Proliferation in type II AECs was reduced during the repair phase in type II AEC-specific HIF-1α knockout mice [74]. The HIF-1α targets, stromal cell-derived factor (SDF) 1 and its receptor, chemokine-X-chemokine receptor (CXCR) 4, were also shown to be up-regulated in type II AECs during lung injury [74]. Finally, SDF1/CXCR4 inhibition impaired type II AEC migration and impaired the restoration of vascular permeability following lung injury [74]. Authors therefore concluded that HIF-1α is activated in type II AECs after lung injury and promotes type II AEC proliferation and spreading during the repair phase [74]. To support the role of HIF-1α in epithelial cell proliferation, Pan et al. showed that PHD1- and PHD3-deficient mice exhibit increased size and number of pulmonary neuroepithelial bodies, along with increased immuno-staining for HIF-1α and the proliferation marker Ki67 [75].

In a study of AEC HIF-2α signaling mentioned above, wild-type mice and mice with HIF-2α deletion in Club and type II AECs were exposed to cobalt or saline [62]. Bronchoalveolar lavage cellularity, inflammatory cytokines, and lung histopathology were assessed [62]. Wild-type mice exhibited a milder recovery from cobalt-induced ALI compared with conditional knockout mice, suggesting a role for AEC-specific HIF-2α in post-ALI repair [62]. The role of HIF-2α in the proliferation of bronchial epithelial cells has also been studied [76]. Torres-Capelli et al. found that bronchial epithelial cell proliferation (mainly in Club/Clara cells) increased in response to hypoxia in vivo, and that this response was dependent upon HIF-2α [76]. However, this proliferative response was not caused by VHL inactivation or inhibition of PHD enzymes, suggesting participation of other oxygen-sensing mechanisms [76]. These findings identified a molecular link between HIF-2α signaling and bronchial epithelial cell proliferation that could be involved in bronchial epithelial cell repair following hypoxia.

### 4.3. HIF Signaling in Leukocytes

Zhu et al. studied the relationship between inflammatory and proliferative properties in alveolar macrophages [77]. In their study, a murine influenza viral pneumonia model was used, and authors found that β-catenin-mediated alveolar macrophage inflammatory activity increased host morbidity, while alveolar macrophage proliferation allowed the repopulation of reparative alveolar macrophages and tissue repair after viral clearance [77]. Activation of Wnt/β-catenin signaling reduced the proliferation of alveolar macrophages and promoted the generation of pro-inflammatory mediators by alveolar macrophages [77]. Wnt signaling also resulted in the formation of a β-catenin-HIF-1α complex in alveolar macrophages, which promoted glycolysis-dependent inflammation and attenuated mitochondrial-metabolism-dependent proliferation after viral infection [77]. The authors then used an HIF-1α activity reporter mouse strain to show that alveolar macrophages with high HIF-1α activity were pro-inflammatory with limited proliferative capacity, while alveolar macrophages with low HIF-1α activity were highly proliferative and expressed genes associated with tissue repair during infection [77]. Inflammatory alveolar macrophages transitioned to healing alveolar macrophages during infection, and the healing alveolar macrophages were necessary for optimal lung repair after viral clearance [77]. Furthermore, Zhu et al. found that Wnt/β-catenin/HIF-1α signaling was conserved in human alveolar macrophages and that HIF-1α signaling promoted macrophage inflammatory activity but inhibited their self-renewal after SARS-CoV-2 infection [77]. This recent study suggests that β-catenin/HIF-1α signaling regulates the inflammatory and reparative activities of lung macrophages, but the role of leukocyte-specific HIF-2 in post-ALI vascular repair is yet to be studied. Opportunities for future research are discussed in the next section.

## 5. Future Perspectives

**Figure 3:**
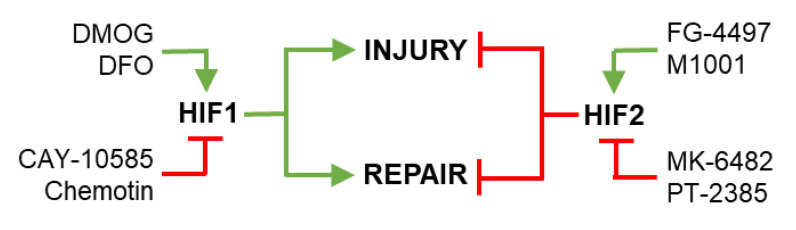
Pharmacological targeting of hypoxia-inducible factor signaling to regulate lung endothelial cell injury and repair. HIF-1 signaling in LECs has been shown to promote (green) LEC injury and repair, while HIF-2 signaling in LECs has been shown to inhibit (red) LEC injury and repair (see Table 1 and main text for references). Upstream of HIF-1 and HIF-2, multiple pharmacological agents have been developed to promote (green) or inhibit (red) the 2 major HIF isoforms. Examples of agents that promote HIF-1 signaling include dimethyloxallyl glycine (DMOG) and deferoxamine (DFO); examples of agents that inhibit HIF-1 signaling include CAY-10585 (Cayman Chemical) and chemotin. Examples of agents that promote HIF-2 signaling include FG-4497 (FibroGen) and M1001; examples of agents that inhibit HIF-2 signaling include MK-6482 (Merck) and PT-2385.

HIF signaling pathways regulate key processes that drive inflammatory lung injury and vascular repair, including LEC viability and junction annealing (Table 1). HIFs could therefore be targeted to reduce vascular injury and/or enhance vascular repair in diseases characterized by inflammatory lung injury, such as severe COVID-19 and ALI/ARDS. FG-4497 is an example of a PHD2 inhibitor that activates HIF-2 signaling, and in turn reduces LEC injury in a mouse model of endotoxemia sepsis [78]. However, the delivery of HIF agonists or antagonists will need to be appropriately timed, given that HIF-1 has been shown to increase LEC injury [45,46] but accelerate repair [66,67], while HIF-2 has been shown to reduce lung injury [48,49,51] but impair repair [72] (Figure 3). Future studies could attempt to develop novel diagnostic tests that identify the phase of injury or repair, thus informing whether injury or repair pathways should be targeted. The aspect of lung injury or vascular repair that the therapy targets should also be considered. For example, some may benefit from therapies that improve LEC or AEC survival or proliferation, while others may benefit from drugs that enhance LEC–LEC junction annealing. Future studies that improve our understanding of the components of inflammatory lung injury and vascular repair will facilitate the development of novel treatments that target specific aspects of lung injury or repair. Additional studies are also required to assess the respective roles of cell- and isoform-specific HIFs in the pathogenesis and repair of severe COVID-19 and ARDS lung injury. For example, the potential role of fibroblast-specific HIF signaling in inflammatory lung injury and repair could be further investigated [79,80].

As a note of caution, the potential side-effects of systemically targeting the HIF signaling pathways could prove to be problematic. For example, while HIF-1 agonism is a plausible therapeutic approach for therapies that aim to accelerate LEC repair, HIF-1-driven increases in angiogenesis and EC proliferation would be undesirable in patients with solid tumors. While it is possible that the agents that promote HIF signaling currently being tested in clinical trials of peripheral arterial disease and anemia [81,82] could be repositioned for the treatment of inflammatory lung injury, it will be vital to monitor off-target side-effects in distant and uninjured tissues. Possible off-target effects of HIF-targeted therapies could be limited by cell- or organ-targeted drug delivery methods [83]. For example, modified nanoparticles and dry powder inhalation drug delivery methods are being developed and tested in our laboratory and the laboratories of others to optimize therapeutic targeting and efficacy against inflammatory lung injury.

## 6. Conclusions

HIFs regulate inflammatory lung injury and vascular repair and therefore represent putative targets for novel therapeutic interventions that aim to treat inflammatory lung conditions such as ALI/ARDS or severe COVID-19. However, in the development of such therapies it will be important to consider HIF isoform and cell specificity, off-target side-effects, and the timing and localization of the therapeutic intervention.

## Figures and Tables

**Table 1:** Regulation of lung endothelial cell injury and repair by hypoxia-inducible factor signaling pathways. HIF target genes in ECs regulate multiple aspects of LEC injury, including EC–EC junction integrity, EC viability and permeability, and EC proliferation and regeneration. For abbreviations, see the main text.

HIF Isoform	Target Gene(s)	LEC Process	Effect	Reference
HIF-1	VE-cadherin	↓ Junction integrity	↑ Injury	[45]
HIF-1	N/A	↓ Cell viability	↑ Injury	[44]
HIF-1	N/A	↑ Cell permeability	↑ Injury	[46]
HIF-1	VEGF	↑ Cell permeability	↑ Injury	[47]
HIF-2	VE-PTP	↑ Junction integrity	↓ Injury	[51]
HIF-2	HGF	↓ EC loss	↓ Injury	[48]
HIF-2	β-catenin	↓ Autophagy	↓ Injury	[49]
HIF-2	N/A	↑ Cell viability	↓ Injury	[50]
HIF-2	Ang1/Tie2/Notch	↑ Cell viability	↓ Injury	[52]
HIF-1	FoxM1	↑ Regeneration	↑ Repair	[66]
HIF-1	Sox17	↑ Regeneration	↑ Repair	[67]
HIF-1	N/A	↑ Regeneration	↑ Repair	[70]
HIF-1	N/A	↑ Regeneration	↑ Repair	[71]
HIF-2	EPCR	↑ Fibrosis	↓ Repair	[72]
